# Editorial: Advances in neuromyelitis optica spectrum disorders

**DOI:** 10.3389/fneur.2022.1005164

**Published:** 2022-08-26

**Authors:** Yu Cai, Jodie M. Burton, Fu-Dong Shi, Wei Qiu, Yangtai Guan

**Affiliations:** ^1^Department of Pharmacology and Experimental Neuroscience, University of Nebraska Medical Center, Omaha, NE, United States; ^2^Department of Clinical Neurosciences, Cumming School of Medicine, University of Calgary, Calgary, AB, Canada; ^3^Department of Clinical Neurosciences and Community Health Sciences, University of Calgary, Calgary, AB, Canada; ^4^Center for Neuroinflammation, Beijing Tiantan Hospital, Capital Medical University, Beijing, China; ^5^China National Clinical Research Center for Neurological Diseases, Beijing, China; ^6^Department of Neurology, Tianjin Neurological Institute, Tianjin Medical University General Hospital, Tianjin, China; ^7^Department of Neurology, Center for Mental and Neurological Disorders and Diseases, The Third Affiliated Hospital of Sun Yat-sen University, Guangzhou, China; ^8^Department of Neurology, Shanghai Jiao Tong University School of Medicine Affiliated Renji Hospital, Shanghai, China

**Keywords:** NMOSD, MOGAD, molecular mechanism, biomarkers, therapy

This topic issues, Advances in Neuromyelitis Optica Spectrum Disorder, jointly published by Frontiers in Neurology and Frontiers in Immunology represents the contribution and efforts of many individuals over the better part of a year. The co-editors of this issue would like to thank the journal staff and all of those who submitted their manuscripts for consideration. We were encouraged to see 40 submissions during the call for papers, with almost half (19) accepted for publication.

This issue was created to highlight important, new work in the field, both in neuromyelitis optica spectrum disorder (NMOSD) and MOG-associated disease (MOGAD), providing new insights into disease mechanisms, diagnosis, and treatment.

## Advances in NMOSD

Formerly known as Devic's disease or neuromyelitis optica (NMO), Neuromyelitis optica spectrum disorders (NMOSD) is an autoimmune demyelinating disease, characterized by demyelination primarily of the optic nerves and spinal cord. The central nervous system (CNS) is more commonly affected in Asian and black patients than in white patients, in whom the age of onset is typically around 40 years (1). Yu et al. reported a higher likelihood of severe disabilities in NMOSD patients with onset after age 40 in central China.

Despite the discovery of autoantibody NMO IgG directed against aquaporin-4 (AQP4), the mechanisms and dynamics of inflammatory degeneration of the CNS in NMOSD, remain elusive. Using a mouse model of spontaneous opticospinal encephalomyelitis (OSE), Petrikowski et al. depicted characteristics of inflammatory and degenerative alterations in both morphology and function of retina and optic nerve with involvement of the complement system. T follicular helper (Tfh) cells derived from germinal center (GC) plays a critical role in promoting pathogenic autoantibody production (2). Cheng et al. demonstrated a correlation of NMOSD recurrence with Tfh cells and CXCL13, in which gut microbiome and bile acid metabolism plays a critical role. These findings lead to the establishment of a gut microbiome–metabolite–Tfh-CXCL13 system to predict the recurrence of NMOSD.

The diagnosis of NMOSD is based on the presence of core clinical manifestation, biomarkers and MRI neuroimaging features (3). AQP4 antibody is a major diagnostic biomarker distinguishing NMOSD from other neuroimmune disorders, but identification of new potential biomarkers also better assist the diagnosis of NMOSD especially in patients with negative AQP4 antibody status (4). Zhang et al. reported a case of NMOSD with similar clinical manifestation of infectious meningomyelitis, highlighting the significant diagnostic and therapeutic importance of biomarker. Based on the expression and clinical correlation analysis, Tang et al. showed the potential of serum repulsive guidance molecules a (RGMa) as a prognostic biomarker of NMOSD. Intriguingly, Shang et al. reported a case of NMOSD with the presence of anti-flotillin, a novel antibody. Kim et al. also review clinical data suggesting glial fibrillary acidic protein (GFAP) as a prospective serum biomarker candidate for NMOSD. Dinoto et al. further summarize current evidence of both CSF and serum biomarkers from the perspectives of antibody titers, cytokine profiles, complement factors and markers of neuronal and astroglial damage. In addition to biomarkers, MRI neuroimaging also plays an indispensable role in diagnosing NMOSD, but it remains a challenge to interpret neuroimaging results for differentiating between neuroimmune disorders in a non-bias manner. Based on the MRI imaging of brain and spinal cord, Huang et al. successfully developed a novel transformer-based deep-learning model for discriminating CNS demyelinating diseases including multiple sclerosis (MS), AQP4-seropositive NMOSD and MOGAD.

Currently, glucocorticoid therapy and adjunctive plasma exchange are the mainstay of the treatment in acute attacks of NMOSD while long-term immunotherapy is recommended to reduce the risk of relapse (5). Deng et al. demonstrated that pregnancy is associated with increased rates of relapse of NMOSD, which can be reduced by prompt immunosuppressive (IS) therapy. Saab et al. reported two cases improved chronic cognitive impairment in AQP4-seropositive NMOSD treated with eculizumab. However, there is still an urgent need for refinement and development of precise therapeutic methods to reduce the rates of long-term disability and mortality while minimizing the adverse effects. Accumulating evidence showed that human umbilical cord mesenchymal stem cells (hUC-MSC) possess differentiation capability, regulate immune response, promote local tissue repair and regeneration, suggesting a therapeutic potential of hUC-MSC in autoimmune disorders (6). Yao et al. thus proposed a study protocol for a prospective multicenter, randomized, placebo-controlled clinical trial for hUC-MSC to treat NMOSD (hUC-MSC-NMOSD).

## Advances in MOGAD

MOGAD, a more recently discovered mimic of NMOSD, albeit one with distinct immunopathological, clinical and treatment features, was a common area of research. Sechi et al. provided a detailed update of clinical and MRI features, as well as diagnostic and treatment guidelines in an international and collaborative effort.

Liu et al. submitted a fascinating look at the immune signatures in a small cohort of RRMS and MOG patients using single cell transcriptome profiling, finding that while CD19+ CXCR4+ naive B cell subsets were expanded in both these MS and MOGAD patients, but in RRMS patients, single-cell transcriptomic was characterized by increased naive CD8+ T cells and cytotoxic memory-like NK cells coupled with a decrease in inflammatory monocytes, while MOGAD exhibited increased inflammatory monocytes and cytotoxic CD8 effector T cells, coupled with decreased plasma cells and memory B cells. Such methods could potentially become a diagnostic tool. Lin et al. presented their work providing evidence that the easily measured plasma C3 and C4 were significantly lower in NMOSD vs. MOGAD and controls, raising the possibility it could be a viable, and easily obtained, biomarker for diagnosis. As is now being understood, SARS-CoV-2 infection can lead to possibly immune mediated complications, including cases of MOGAD and NMOSD. Matsumoto et al. provided a fascinating and complex case of such a patient to educate us in recognizing and considering this possibility. Furthermore, Di Pauli et al. review the co-occurrence of MOG and AQP-4 antibodies with another common virus, VZV, finding both are, rare, MOG rarer still.

Neuroimaging also serves as one of the most critical diagnostic methods for MOGAD. Wang et al. showed 4 cases of MOGAD with abnormal signals in cortical or brainstem observed in cranial MRI. Rechtman et al. demonstrated an association between a relapsing disease course and reduced volume in gray matter in cerebrum and hippocampus within the 1st year of diagnosis.

In conclusion, this Research Topic attempts to show the most up-to-date advances in the field of NMOSD and MOGAD, covering a broad range of topics from mechanistic studies, case reports, diagnostic innovations and therapeutic strategies. We hope that these findings could provide insights for both physicians and researchers interested in the CNS demyelinating disorders.

## Author contributions

All authors listed have made a substantial, direct, and intellectual contribution to the work and approved it for publication.

## Conflict of interest

The authors declare that the research was conducted in the absence of any commercial or financial relationships that could be construed as a potential conflict of interest.

## Publisher's note

All claims expressed in this article are solely those of the authors and do not necessarily represent those of their affiliated organizations, or those of the publisher, the editors and the reviewers. Any product that may be evaluated in this article, or claim that may be made by its manufacturer, is not guaranteed or endorsed by the publisher.
